# Prevalence of celebrity worship: Development and application of the short version of the Celebrity Attitude Scale (CAS-7) on a large-scale representative sample

**DOI:** 10.1556/2006.2024.00019

**Published:** 2024-04-18

**Authors:** Ágnes Zsila, Lynn E. McCutcheon, Rita Horváth, Róbert Urbán, Borbála Paksi, Gergely Darnai, József Janszky, Zsolt Demetrovics

**Affiliations:** 1 Institute of Psychology, Pázmány Péter Catholic University, Budapest, Hungary; 2 Institute of Psychology, ELTE Eötvös Loránd University, Budapest, Hungary; 3 North American Journal of Psychology, Winter Garden, FL, USA; 4 Doctoral School of Psychology, ELTE Eötvös Loránd University, Budapest, Hungary; 5 Institute of Education, ELTE Eötvös Loránd University, Budapest, Hungary; 6 Department of Behavioural Sciences, Medical School, University of Pécs, Pécs, Hungary; 7 Department of Neurology, Medical School, University of Pécs, Pécs, Hungary; 8 HUN-REN-PTE Clinical Neuroscience MR Research Group, Pécs, Hungary; 9 Centre of Excellence in Responsible Gaming, University of Gibraltar, Gibraltar, Gibraltar; 10 College of Education, Psychology and Social Work, Flinders University, Adelaide, Australia

**Keywords:** celebrity worship, prevalence, scale development, structural equation modeling

## Abstract

**Background and aims:**

Celebrity worship, defined as an excessive admiration towards celebrities, has generated considerable research and public interest. A widely used assessment instrument to measure celebrity worship is the 23-item Celebrity Attitude Scale. However, concerns have been raised regarding the measurement, including the inconsistent factor structure and lack of a cut-off point to identify “celebrity worshipers”. The present study aims to address these concerns by testing the psychometric appropriateness of a short, 7-item version of the CAS (i.e., CAS-7) and estimating the prevalence of individuals with high-level celebrity admiration using a representative sample of Hungarian adults (between 18 and 64 years of age) according to gender, age, geographic location, and size of residence.

**Methods:**

The total sample comprised 2028 respondents, of which 769 valid responses were administered from participants who reported having a favorite celebrity (51.11% men, *M*_*age*_ = 36.38 years, *SD* = 13.36).

**Results:**

Results indicated an excellent model fit for the two-factor and bifactor model of the CAS-7. Based on the suggested cut-off score of 26, the prevalence of high-level celebrity admiration is 4.53% in the Hungarian adult population (18–64 years of age) and 8.51% among young adults (18–34 years of age). Individuals with this high level of admiration towards a favorite celebrity reported more symptoms of problematic Internet use, depression, anxiety, and stress than individuals with general celebrity admiration levels.

**Discussion and conclusions:**

The CAS-7 demonstrated sound psychometric properties, confirming its applicability in research and practice.

## Introduction

Admiration towards celebrities, termed as celebrity worship, has been extensively researched in the past two decades (Brooks, 2021). Celebrity worship represents excessive forms of attraction towards a favorite celebrity, which are generally assessed using the Celebrity Attitude Scale (CAS; McCutcheon, Lange, & Houran, 2002, 2004). The construction of the CAS was based on previous measures assessing the parasocial phenomena (Rubin et al., 1985a, 1985b; Stever, 1991) and motivations for participating in leisure activities (e.g., sports; Wann, 1995). Although more than 100 published studies have used the CAS to date (Locker et al., 2023), concerns have been raised regarding the measurement, involving the inconsistent findings regarding the factor structure of the CAS and the lack of a cut-off score to identify individuals with an excessive admiration towards celebrities (i.e., “celebrity worshipers”; McCutcheon & Aruguete, 2021). To date, no studies have been conducted using a representative sample to estimate prevalence rates for “celebrity worshipers”, either. This study endeavors to fill these gaps.

The Absorption–Addiction model provides a theoretical basis for celebrity worship, which proposes that many persons become interested in celebrities because they are a frequent source of entertainment that can be discussed with friends, while a smaller proportion of individuals become intensely involved in the personal lives of their favorite celebrities (McCutcheon et al., 2002, 2004). This progressive model is represented by different dimensions of the CAS. The first dimension, “Entertainment–Social” (ES), involves healthy fan activities such as following news about a favorite celebrity or enjoying the company of others with a similar interest. The second dimension, termed “Intense–Personal” (IP), refers to excessive feelings toward the celebrity (e.g., considering the favorite celebrity as a soul mate), while the third dimension, “Borderline–Pathological” (BP), comprises problematic, compulsive behaviors such as feeling urged to learn the personal habits of the favorite celebrity (McCutcheon et al., 2002).

Although the most commonly used version is the 23-item CAS (Brooks, 2021), some studies investigated the factor structure of a 34-item and a 22-item version (Maltby, Day, McCutcheon, Gillett, et al., 2004; Maltby, Day, McCutcheon, Houran, & Ashe, 2006). Most of these studies found evidence for the original three-factor structure (e.g., Maltby, Day, McCutcheon, Martin, & Cayanus, 2004; Maltby, Giles, Barber, & McCutcheon, 2005), although the number and content of items comprising the respective factors were inconsistent (see Maltby et al., 2005; North, Sheridan, Maltby, & Gillett, 2007). Moreover, early studies indicated an essentially unidimensional structure, suggesting that a total score can also be calculated as an indicator of general admiration towards a favorite celebrity (Maltby & McCutcheon, 2001; McCutcheon et al., 2002). However, decades of evidence shows that higher scores on the IP and BP subscales are associated with clinical features (e.g., symptoms of depression, anxiety, and dissociation), while scores on the ES factor are largely unrelated or weakly related to mental health problems (Brooks, 2021). Based on the convergent associations with clinical constructs across IP and BP, and also considering that more recent studies have reported high correlations (above 0.80) between these factors (e.g., Aruguete, Huynh, Collisson, McCutcheon, & Piotrowski, 2020; Martinez-Berman, McCutcheon, & Huynh, 2021), this study tests the psychometric appropriateness of a unidimensional, two-, three- and bifactor structure of the CAS.

Recent studies emphasize the need for short, even single-item assessment instruments, which can assist researchers and practitioners with limited resources and administration time of a questionnaire (Bőthe, Tóth-Király, Demetrovics, & Orosz, 2021; Bőthe, Vaillancourt-Morel, Dion, Štulhofer, & Bergeron, 2021; Danner, Treiber, & Bosnjak, 2019). Therefore, this study tests the psychometric appropriateness of a short, 7-item version of the CAS, which could facilitate an easy administration of celebrity admiration levels.

To test the convergent validity of the brief CAS, the association of this measure with two assessment instruments will be examined which assess similar constructs (i.e., wishful identification and parasocial relationship). Wishful identification refers to the wish to become or behave like a favorite fictional character or a living media personae (Hoffner & Buchanan, 2005), and is assessed by a 5-item self-report measure developed by Hoffner and Buchanan (2005). A parasocial relationship is a long-term, traditionally one-sided, illusory emotional bond between a media personae and the media user (e.g., the follower perceives the influencer as a natural, down-to-earth person who could be a friend; Horton & Richard Wohl, 1956), which is commonly assessed by the Parasocial Interaction Scale, for which 20-item, 15-item and 8-item versions are available (PSI-Scale; Rubin et al., 1985a, 1985b; Rubin & Perse, 1987).

Celebrity worship has been associated with psychological difficulties such as symptoms of depression and anxiety (see Sansone & Sansone, 2014), and problematic Internet use (Zsila, McCutcheon, & Demetrovics, 2018). Therefore, the association of these correlates with the 7-item CAS will also be investigated in this study to further validate this brief measure.

Stever (2011) also expressed the need for a cut-off point, which can differentiate between celebrity worshipers (i.e., individuals showing excessive levels of celebrity admiration) and non-worshipers. McCutcheon et al. (2002, p. 80) suggested that “sufficiently high levels of celebrity worship invariably lead to signs of pathology”; however, no exact cut-off threshold was proposed. Although some studies attempted to estimate prevalence rates using theoretical midpoints on the CAS (Maltby, Houran, & McCutcheon, 2003; McCutcheon & Aruguete, 2021; Stever, 2011), a consensual cut-off threshold is still lacking. A cut-off point which allows for the identification and screening of individuals with generally high-level celebrity admiration would considerably contribute to the clinical assessment of celebrity worship tendencies (Stever, 2011). Finally, Stever (2011) pointed out that studies using the CAS have often used student samples and specific fan community members, which limits the generalizability of findings. This study finally provides a prevalence rate for individuals showing high-level celebrity admiration in a representative sample of Hungarian adults, thereby extending the generalizability of findings.

### Aim of the study

Overall, the present study aims to fill some major gaps in the measurement of celebrity worship by 1) testing the psychometric appropriateness of a brief, 7-item version of the CAS; 2) investigating multiple theoretical factor structures to identify the best-fitting model; 3) estimating a cut-off score to identify individuals with high-level celebrity admiration (i.e., “celebrity worshipers”), and 4) estimating a prevalence rate for individuals with high-level celebrity admiration in the general population of Hungarian adults. Constructing a brief, psychometrically sound assessment instrument to measure celebrity worship, which is also appropriate to identify individuals with high levels of attraction towards their favorite celebrities can facilitate the assessment of celebrity worship in future research. Furthermore, this study is the first to provide a prevalence rate for high-level celebrity admiration in the general population using a large-scale representative sample of Hungarian adults.

## Methods

### Participants and procedure

Target population of the present research was the general population of Hungary aged between 18 and 64 years. Participants were selected by quota sampling, stratified by gender, age group (18–24, 25–34, 35–44, 45–54, 55–64 years old), geographic location (19 counties and Budapest) and residence (county seats, town of county rank, other town, large village, municipality). The population of young adults aged 18–34 years, who are highly affected in terms of the target variables under investigation, was oversampling to provide an individual sample size of 1,000 participants. With this sample size, at a 95% confidence level, the extent of the theoretical margin of error in the sample of adults aged 18–64 years was ±2.2%, and ±3.1% in the sample of young adults aged 18–34 years. Therefore, the total sample comprised 2,028 participants. Computer-assisted personal interviewing (CAPI) technique was applied by professional interviewers.

Of the 2,028 individuals, 800 participants reported having a favorite celebrity (39%) after reading McCutcheon et al.’s (2004) general definition of a favorite celebrity. These individuals comprised the target population of this specific study, which focused on the phenomenon of celebrity admiration. Respondents who provided invalid answers based on the definition of a favorite celebrity (e.g., a person who died long years ago such as Napoleon; *n* = 18), or did not respond to at least 5 items of the present 7-item CAS (*n* = 13) were excluded from further analysis. Therefore, the final sample comprised 769 participants who all reported having a favorite celebrity (51.11% men, *M*_*age*_ = 36.38 years, *SD* = 13.36, age range: 18–63 years). Most participants had at least a secondary school certificate (69.31%), while 14.43% obtained a BA or MA diploma, and 16.25% received primary school education. Participants selected mostly musicians (43.69%), actors (23.02%), and athletes (20.16%) as favorite celebrities.

### Measures

A 7-item version of the CAS (McCutcheon et al., 2002, 2004) was used to assess celebrity admiration levels (see items in Table 1). Items were selected from the commonly used 23-item version of the CAS (McCutcheon, Maltby, Houran, & Ashe, 2004), based on both theoretical and statistical considerations (see a detailed item selection protocol in SM1 in Supplementary material). Regarding theoretical considerations, core components (i.e., key terms) of each dimension based on the concept by McCutcheon et al. (2004) were considered in the item content with the greatest variability. Relating to statistical considerations, items with consistently high factor loadings in their respective factor and those without conflicting results (e.g., positive and negative factor loadings across the IP and BP dimensions) were selected for further consideration based on previous studies (e.g., Maltby et al., 2005; Wong, McCutcheon, Rodefer, & Carter, 2023). Overall, 3 of 10 items of the ES, 2 of 9 items of the IP, and 2 of 4 items of the BP subscale were included in the short, 7-item version of the CAS (CAS-7, see Table 1). Items were rated on a 5-point Likert-scale (1 = strongly disagree, 5 = strongly agree).

**Table 1.** Item content of the 7-item version of the CAS (CAS-7)

Item number	Item content	Indicator in further data analysis
1	I share with my favorite celebrity a special bond that cannot be described in words.	IP2
2	It is enjoyable just to be with others who like my favorite celebrity.	ES1
3	I often feel compelled to learn the personal habits of my favorite celebrity.	BP2
4	I like watching and hearing about my favorite celebrity when I am in a large group of people.	ES2
5	When something good happens to my favorite celebrity I feel like it happened to me.	IP1
6	Keeping up with news about my favorite celebrity is an entertaining pastime.	ES3
7	If someone gave me several thousand dollars to do with as I please, I would consider spending it on a personal possession (like a napkin or paper plate) once used by my favorite celebrity.	BP1

^a^Instruction: Please indicate the extent to which you agree or disagree with the following statements. 5 = strongly agree; 4 = agree; 3 = uncertain or neutral; 2 = disagree; 1 = strongly disagree.

^b^ES = Entertainment–Social, IP = Intense–Personal, BP = Borderline–Pathological dimension.

The 8-item Parasocial Interaction Scale (PSI-Scale; Rubin & Perse, 1987) was used to assess the strength of parasocial relationship with a favorite celebrity. Items were rated on a 5-point Likert-scale (1 = strongly disagree, 5 = strongly agree). Higher scores indicated stronger parasocial relationship (*α* = 0.86).

Wishful identification was assessed using the 5-item measure by Hoffner and Buchanan (2005), which measured participants' wish to identify with their favorite celebrity using a 5-point Likert-scale (1 = strongly disagree, 5 = strongly agree). Higher scores indicated stronger wishful identification (*α* = 0.82).

Depression, anxiety, and stress levels were assessed using the 9-item Depression, Anxiety, Stress Scale (DASS-9; Yusoff, 2013). Items were rated on a 4-point scale (0 = did not apply to me at all, 3 = applied to me very much, or most of the time). As correlations between subscales were high (*r*s ranged between 0.72 and 0.75), a total score was used in further analysis (*α* = 0.91), following the protocol by Yusoff (2013).

Symptoms of problematic Internet use were assessed by the 9-item Problematic Internet Use Questionnaire (PIUQ-9; Koronczai et al., 2011). Items were rated on a 5-point scale (1 = never, 5 = almost always). Higher scores indicated more symptoms of problematic Internet use (*α* = 0.93).

### Statistical analysis

Descriptive statistics, group comparisons, and correlations were calculated using SPSS 21.0, while structural equation modeling (SEM) was performed using Mplus 7.4 (Muthén & Muthén, 1998-2017). To explore the psychometric properties of the 1-, 2-, 3-, and bifactor structure of the CAS-7, confirmatory factor analysis (CFA) was conducted using a robust maximum likelihood estimator (MLR). The Yuan-Bentler T2* test statistic was used to compare model fit indices (Muthén & Muthén, 1998-2017). To identify individuals with high-level celebrity admiration (“celebrity worshipers”), latent profile analysis (LPA) was conducted on the CAS-7. The final number of classes of the 1–5 tested models was selected based on the following fit indices: lower scores on the Akaike information criterion (AIC), bias-corrected Akaike information criterion (CAIC), Bayesian information criterion (BIC), and sample-size adjusted Bayesian information criterion (SSABIC); values close to 1 regarding entropy; and the Lo-Mendell-Rubin Adjusted Likelihood Ratio Test (LMR Test) with a non-significant *p*-value (*p* > 0.05) indicating the adequacy of a model with one fewer class (Muthén & Muthén, 1998-2017). Based on the classes from the LPA, sensitivity, specificity, positive (PPV) and negative predictive values (NPV), and potential cut-off thresholds were estimated (Altman & Bland, 1994; Glaros & Kline, 1988). To investigate possible correlates of the CAS-7, a multiple indicators multiple outcomes (MIMIC) model was constructed in which CAS factors were defined as outcomes, while major demographics, depression, anxiety, and stress, and problematic Internet use were predictors. The following fit indices were considered for the CFA and MIMIC models (Bentler, 1990; Brown, 2015): comparative fit index (CFI; ≥0.95 for good, ≥0.90 for acceptable), Tucker-Lewis index (TLI; ≥0.95 for good, ≥0.90 for acceptable), the root-mean-square error of approximation (RMSEA; ≤0.06 for good, ≤0.08 for acceptable) with its 90% confidence interval (CI), and the standardized root-mean-square residuals (SRMR; ≤0.05 for good, ≤0.10 for acceptable). Missing data (less than 5% for all variables) were handled using full information maximum likelihood (FIML), which is the default when using an MLR estimator in Mplus.

Finally, prevalence rate estimations were performed on weighted samples. To counterbalance the oversampling of young adults (aged 18–34 years) and minor distortions occurring during data collection in the population of 18–64 years old, a non-count preserving matrix weighting based on stratification categories (gender, age, geographic location, and size of residence) was applied for the whole sample (18–64 years old) (*N*_weighted_ = 2,000). Leveraging the advantages of the oversampling of the young adult population (i.e., individuals with potentially higher involvement), prevalence rates are reported separately for the young adult population. Again, minor distortions that occurred during data collection were counterbalanced by weighting, resulting in a weighted sample size of *N*_weighted_ = 1,001. To eliminate the potential risk of distortion in the associations due to weighting, analyses other than the prevalence estimations, were conducted on the raw, unweighted database.

### Ethics

The present study was conducted in accordance with the Declaration of Helsinki. Ethical approval was granted by the Institutional Review Board of the Institute of Psychology, Pázmány Péter Catholic University (protocol number: 2023_69). All participants were informed about the study's aim and provided informed consent.

## Results

### Factor structure of the CAS-7

**Fig. 1.**
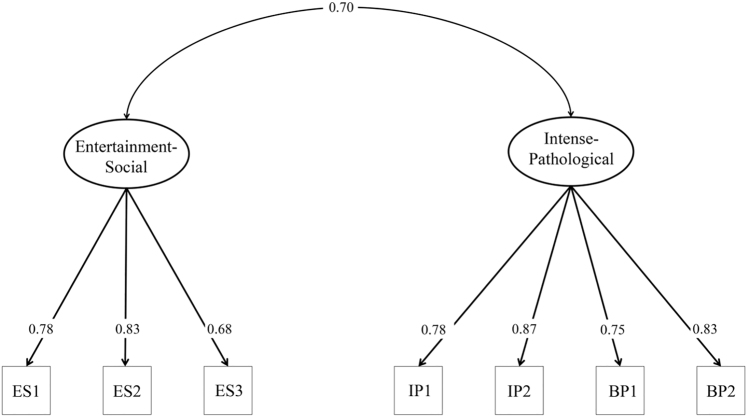
Two-factor model of the CAS-7. ^a^Latent variables are represented in oval, while observed variables are represented in squares. ^b^ES = Entertainment–Social; IP = Intense–Personal, BP = Borderline–Pathological. Item numbers and their content are presented in Table 1. ^c^Standardized factor loadings are represented on single-headed arrows (*p* < 0.001). ^d^Correlation coefficient for the association between latent variables is presented on the double-headed arrow (*p* < 0.001). Cronbach's α = 0.81 for ES and α = 0.88 for IPBP

**Fig. 2.**
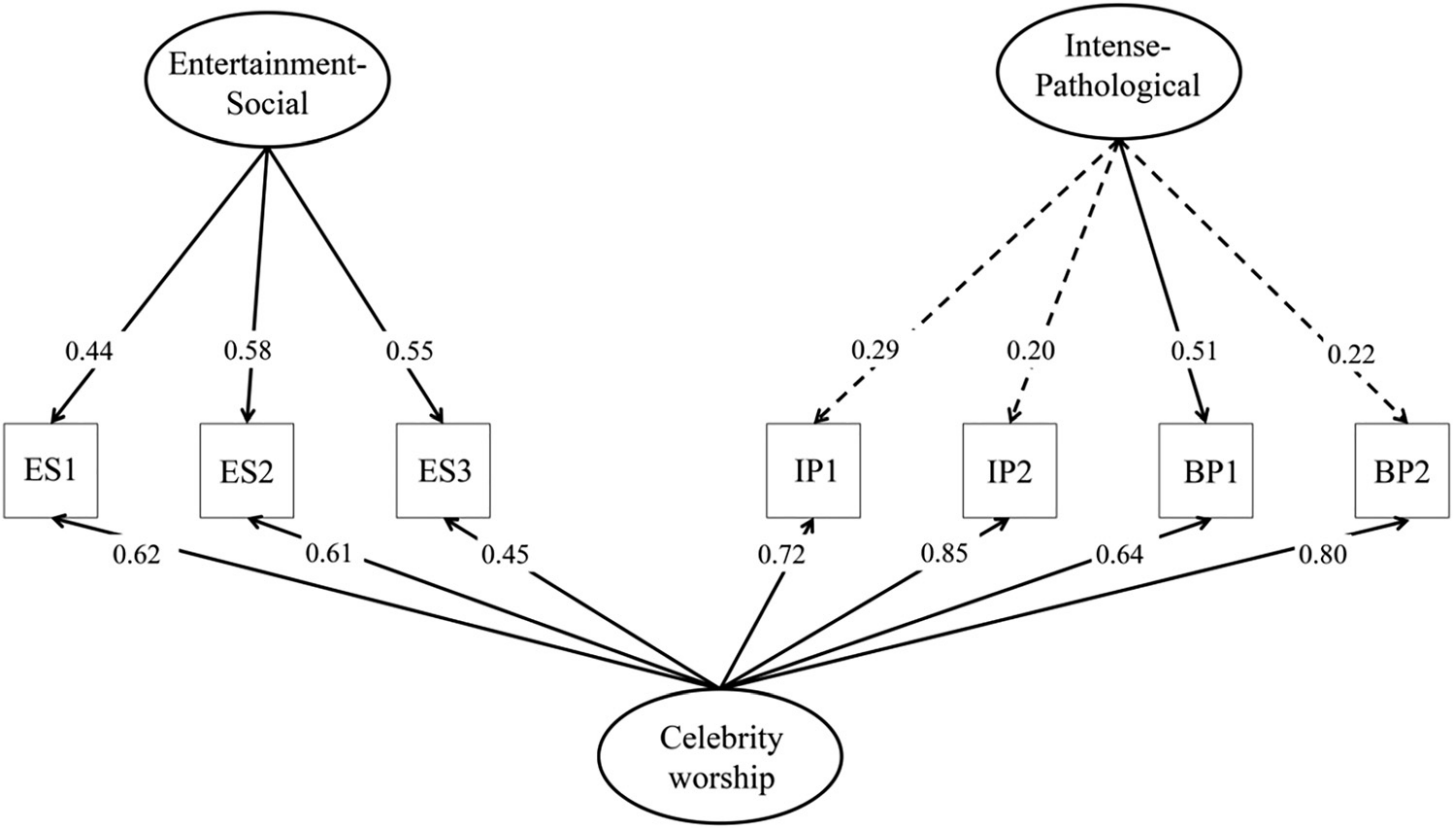
Bifactor model of the CAS-7. ^a^Latent variables are represented in oval, while observed variables are represented in squares. ^b^ES = Entertainment–Social; IP = Intense–Personal, BP = Borderline–Pathological. ^c^Item numbers and their content are presented in Table 1. ^d^Standardized factor loadings are represented on single-headed arrows (*p* < 0.001). Dashed arrows represent nonsignificant regression paths (*p* > 0.05). ^e^Correlations among specific factors (i.e., ES and IPBP) and the general factor (CW) with specific factors were fixed to 0. ^f^Reliability indices for multidimensional constructs were calculated for the general factor (ECV = 0.72, Ω = 0.91, Ω hierarchical = 0.77, H = 0.88), ES (ECV = 0.18, Ω = 0.81, Ω hierarchical = 0.38, H = 0.54), and IPBP (ECV = 0.10, Ω = 0.89, Ω hierarchical = 0.13, H = 0.35). ^g^Cronbach's alpha for the CW general factor was 0.88

Based on prior studies (Maltby, Houran, Lange, Ashe, & McCutcheon, 2002, 2005; McCutcheon et al., 2002), multiple factor structures were tested on the CAS-7 items. Specifically, 1-, 2-, 3-, and bifactor models were investigated using CFA. According to the model fit indices (see Table 2), the 1-factor model representing celebrity worship as a latent construct did not fit the data (Fig. S1). The 2-factor model with two intercorrelated celebrity worship factors (i.e., ES and IPBP) showed an excellent fit with high internal consistency (Fig. 1). The IPBP factor comprised items from the IP and BP theoretical factors. The correlation between ES and IPBP latent factors was high. The 3-factor model also showed good fit to the data with high reliability indices of the specific factors; however, the correlation between IP and BP latent factors was extremely high, indicating severe overlap between the two constructs (Fig. S2). Therefore, the 2-factor structure was considered as a basis for the final, bifactor model. The bifactor model yielded excellent fit. The bifactor model yielded closer fit to the data than the 2-factor model (Δ*χ*^*2*^ = 22.336, Δ*df* = 6, *p* = 0.001), Reliability indices (Fig. 2) indicate that the contribution of specific factors, especially the IPBP to the total variance is modest in comparison with the general factors' contribution. Therefore, the construct underlying the CAS-7 can be considered as essentially unidimensional with some multidimensionality. Estimating a total score (*M* = 18.20, *SD* = 6.81, range: 7–35, skewness = 0.20, kurtosis = −0.61) and scores for the ES (*M* = 9.23, *SD* = 3.35, range: 3–15, skewness = −0.23, kurtosis = −0.76) and IPBP (*M* = 9.00, *SD* = 4.33, range: 4–20, skewness = 0.50, kurtosis = −0.74) dimensions can also be meaningful. Inter-item correlations ranged between 0.24 and 0.72, *M*_*r*_ = 0.50).

**Table 2.** Fit indices of factor structures tested for the CAS-7

Model	df	χ^2^	CFI	TLI	RMSEA [ 90% CI]	SRMR
1-factor	14	281.483*	0.863	0.794	0.158 [0.142–0.174]	0.078
2-factor	13	36.401*	0.988	0.981	0.048 [0.030–0.067]	0.029
3-factor	11	37.417*	0.986	0.974	0.056 [0.037–0.076]	0.028
Bifactor	7	12.714	0.997	0.991	0.033 [0.000–0.061]	0.011

^a^*p* < 0.001.

### Correlates of celebrity worship

First, confirming the validity of the CAS-7, the associations of two theoretically similar constructs with celebrity worship were investigated. Celebrity admiration (i.e., the total score of the CAS-7) showed a positive and moderate association with parasocial relationship (*r* = 0.45, *p* < 0.001) and wishful identification (*r* = 0.51, *p* < 0.001). Second, a MIMIC model was constructed to explore convergent and divergent associations of celebrity worship dimensions with demographics and mental health indicators (Table 3). Lower age predicted generally higher celebrity admiration, while lower educational level predicted higher scores on the IPBP factor. However, demographics explained only a negligible proportion of the total variance of celebrity worship (below 5%). Symptoms of problematic Internet use, depression, anxiety, and stress predicted generally higher celebrity admiration. 95% CIs were overlapping for both the DASS (*β* = 0.10 [0.02; 0.19] for ES, *β* = 0.27 [0.18; 0.36] for IPBP) and the PIUQ (*β* = 0.14 [0.05; 0.23] for ES, *β* = 0.25 [0.16; 0.33] for IPBP), indicating no significant difference in the strength of associations between celebrity admiration and mental health indicators across ES and IPBP.

**Table 3.** Multiple Indicators Multiple Causes (MIMIC) model predicting celebrity admiration levels

Predictor variables	Outcome variables (*β, SE*)	
	Entertainment–Social	Intense–Pathological
**Model I**		
Gender	−0.070 (0.040)	−0.025 (0.038)
Age	−0.151 (0.038)***	−0.117 (0.037)**
Educational level	−0.065 (0.039)	−0.109 (0.039)*
***R*** ^***2***^	3.2%	2.6%
**Model II**		
Gender	−0.059 (0.039)	−0.003 (0.035)
Age	−0.132 (0.039)**	−0.085 (0.034)*
Educational level	−0.050 (0.038)	−0.079 (0.035)*
Depression, anxiety, and stress	0.104 (0.044)*	0.269 (0.044)***
Problematic Internet use	0.140 (0.046)**	0.247 (0.044)***
***R*** ^***2***^	6.8%	21.0%

^a^***p < 0.001; **p < 0.01; *p < 0.05.

^b^Gender (1 = man, 2 = woman) and educational level (1 = primary school education, 2 = secondary school certificate or BA/MA diploma) were binary variables.

^c^In Model I, observed demographic variables were entered in the model, while in Step II, mental health indices as latent variables were added.

^d^Fit indices for the models are presented in Table S1.

### Determining a cut-off score to identify high-level celebrity admiration

**Fig. 3.**
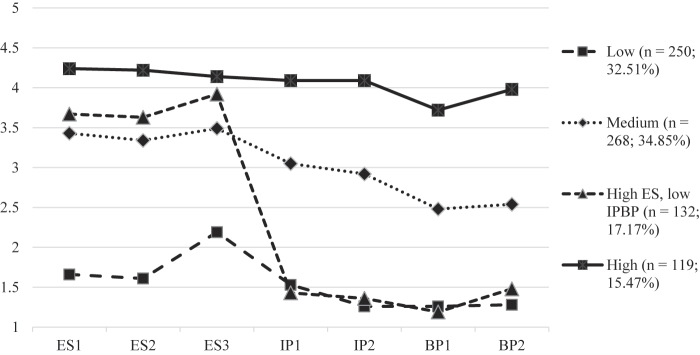
Item scores on the CAS-7 across the four latent classes

Based on theoretical (i.e., the Absorption-Addiction model; McCutcheon et al., 2002, 2004) and statistical considerations (i.e., high inter-factor correlations, convergent predictors for the ES and IPBP subscales, and a strong general factor in the bifactor model structure), LPA was performed using all items of the CAS-7. Based on the fit indices (Table S2), the 4-class model was selected in which the first class (32.51%) represented individuals with low-level celebrity admiration, while the second class (34.85%) comprised individuals with medium-level celebrity admiration. The third class (17.17%) included individuals scoring high on all ES items, while scoring low on all IPBP items. Finally, the fourth class (15.47%) represented individuals scoring high on all CAS-7 items (see Fig. 3). Differences in mental health indicators among classes are presented in Table S3.

The fourth class was used as the “gold standard” for subsequent calculations of sensitivity, specificity, PPV, NPV, and accuracy of classification based on possible cut-off scores (Table 4). The score of 26 was found to be an optimal cut-off point to identify individuals with high-level celebrity admiration (“celebrity worshipers”) with high accuracy (97.7%). Using this cut-off score, the vast majority of individuals with high-level celebrity admiration (93.9%) and general, mild celebrity admiration (98.3%) were identified correctly. Based on the cut-off point of 26, 14.95% of individuals (*n* = 115) could be considered to have high-level celebrity admiration in this subsample of individuals with a favorite celebrity. These individuals were slightly younger, less educated, and reported more symptoms of problematic Internet use, depression, anxiety, and stress compared to individuals with general celebrity admiration levels (see Table 5).

**Table 4.** Cut-off thresholds for the CAS-7

Cut-off score	True positive	True negative	False positive	False negative	Sensitivity (%)	Specificity (%)	PPV (%)	NPV (%)	Accuracy (%)
17	119	330	320	0	100	50.8	27.1	100	58.4
18	119	367	283	0	100	56.5	29.6	100	63.2
19	119	399	251	0	100	61.4	32.2	100	67.4
20	119	436	212	0	100	67.3	36.0	100	72.4
21	119	469	181	0	100	72.2	39.7	100	76.5
22	119	514	136	0	100	79.1	46.7	100	82.3
23	119	552	98	0	100	84.9	54.8	100	87.3
24	118	585	64	1	99.2	90.1	64.8	99.8	91.5
25	118	620	30	1	99.2	95.4	79.7	99.8	96.0
**26**	**108**	**643**	**7**	**11**	**90.8**	**98.9**	**93.9**	**98.3**	**97.7**
27	84	649	1	35	70.6	99.9	98.8	94.9	95.3
28	65	650	0	54	54.6	100	100	92.3	93.0

^a^PPV = positive predictive value; NPV = negative predictive value.

^b^The suggested cut-off threshold is marked in bold.

^c^Scores on the short version of the Celebrity Attitude Scale (CAS-7) can range from 7 to 35.

**Table 5.** Group comparisons across individuals with general and high-level celebrity admiration based on the 26-point cut-off on the CAS-7

Variables	Groups		Estimates	
	Individuals with general celebrity admiration (*n* = 654)	Individuals with high-level celebrity admiration (*n* = 115)	χ^2^/*t*	Effect size (odd ratio/*d)*
Gender – men (*n*, %)	331 (50.61%)	62 (53.91%)	0.43	1.14
Age (*M, SD*)	37.09 (13.42)	32.35 (12.34)	3.75***	0.37
Educational level – primary school (*n*, %)	97 (15.04%)	28 (24.35%)	5.94*	1.85
Depression, anxiety, and stress (*M, SD*)	1.16 (1.49)	1.83 (1.90)	−3.62***	0.39
Problematic Internet use (*M, SD*)	12.98 (5.46)	16.91 (7.53)	−5.23***	0.60

^a^***p < 0.001; *p < 0.05.

^b^Binary logistic regression results with odds ratios are reported for gender and educational level, while *t*-test results and Cohen *d* are reported for all other variables.

Finally, prevalence rates were estimated on the weighted sample of Hungarian adults between 18 and 64 years (*N* = 2,000), and subsequently, on the subsample of the 18–34 years old population (*N* = 1,001). The prevalence rates for high-level celebrity admiration (i.e., “celebrity worshipers”) are 4.53% for the adult population (18–64 years old), and 8.51% for the young adult population (18–34 years old).

## Discussion

Decades of evidence suggest that higher levels of celebrity admiration are associated with poorer mental health (Brooks, 2021), which underlines the need for accurate measurement to assist in screening problematic levels of celebrity worship in research and practice. This study investigated the psychometric properties of a short, 7-item version of the Celebrity Attitude Scale (CAS; McCutcheon et al., 2002, 2004) and determined a cut-off score, which allows for the distinction between general and high-level celebrity admiration. Findings supported the psychometric appropriateness of the CAS-7 for research and screening purposes. Moreover, establishing the psychometric appropriateness of this short measure and the representative sample of Hungarian adults allowed for the estimation of prevalence rates of high-level celebrity admiration in the general population. It was found that 4.53% of Hungarian adults can be considered as having high-level celebrity admiration (i.e., “celebrity worshipers”) that could be associated with higher risk for experiencing mental health problems. The prevalence among young adults aged 18–34 years was much higher (8.51%).

Regarding the factor structure of the short, 7-item CAS (i.e., the CAS-7), the 2-factor and bifactor models yielded excellent fit with high internal consistency and construct validity. Results also indicated that the CAS-7 is largely unidimensional. However, the unidimensional structure did not fit the data, and the bifactor model also indicated some multidimensionality, which can be explained by two latent factors (i.e., Entertainment–Social [ES] and a comprehensive factor merging the Intense–Personal [IP] and Borderline–Pathological [BP] dimensions). The high association between IP and BP subscales in this study was in line with the findings of recent studies reporting similarly strong correlations between the two problematic dimensions (see Aruguete et al., 2020; Martinez-Berman et al., 2021), suggesting a considerable overlap between these constructs. Confirming this assumption, several studies found convergent associations with mental health indicators across IP and BP (see Sansone & Sansone, 2014 for a review). The comprehensive factor of IP and BP was termed “Intense–Pathological” (IPBP) in this study as an indicator of “Intense” feelings towards the admired celebrity and “Pathological” behavioral patterns.

Similar to the use of the 23-item CAS, calculation of a total score and scores for the specific subscales are both meaningful and interpretable when using the CAS-7. Therefore, similar to the 23-item CAS, the CAS-7 is an appropriate tool (1) to investigate the differences in quality between nonproblematic (ES) and problematic (IPBP) dimensions and their possibly convergent and divergent associations with various psychological constructs (e.g., personality variables, mental health indicators), and (2) to gain a global picture of a person's celebrity admiration level. Moreover, a cut-off point of 26 was determined to distinguish between general and high-level celebrity admiration. Based on this cut-off, 15% of participants could be considered as individuals with high-level celebrity admiration in the final sample of those who reported having a favorite celebrity. Using theoretical midpoints, Stever (2011) found that 12% and 49% scored high on ES, 4% and 12% on IP, and 1% and 5% on BP among fan community members. Maltby and Day (2011) also reported that 15.1% and 22.8% of adults scored high on ES, 5.1% and 8% on IP, and 1.9% and 2.5% on BP. The present proportion of individuals with high-level celebrity admiration is somewhat similar to those found in relation to the ES subscale in previous studies among fan community members and undergraduates. This result aligns with the current findings regarding the essentially unidimensional nature of the CAS-7 in which the contribution of ES items to the general factor (i.e., celebrity worship) was notable. This finding also corresponds to the theoretical framework of celebrity worship. Specifically, the Absorption–Addiction model (McCutcheon et al., 2002, 2004) suggests that a person showing an excessive admiration towards a celebrity (i.e., scoring high on IP and BP) should also be interested in the celebrity and derive gratification from the fan activity (i.e., scoring high on ES).

In the general population of Hungarian adults, the prevalence of high-level celebrity admiration was 4.53%. This rate was higher among young adults aged 18–34 years (8.51%), indicating that celebrity admiration is more prevalent among younger adults. To date, these prevalence rates are the first estimates of high-level celebrity admiration (i.e., “celebrity worshipers”) in a general population sample.

The present results indicated weak, positive associations of mental health indicators with ES and IPBP, which is in line with the findings by Maltby et al. (2003), indicating that celebrity worship, even for ES purposes is associated with poorer mental health. However, several studies found associations between poorer mental health and the two problematic dimensions (i.e., IP and BP), while mental health was unrelated to ES (Maltby et al., 2006; Sansone & Sansone, 2014). As the scope of mental health indicators was narrow in this study, future research is needed to investigate convergent and divergent predictors of ES and IPBP using a broader range of indicators.

The present findings provide support for the recommendation of McCutcheon et al. (2002) that high levels of general celebrity worship are associated with an increased risk of psychopathology. Indeed, individuals with high-level celebrity admiration reported more symptoms of problematic Internet use, depression, anxiety, and stress than individuals with general celebrity admiration levels in this study. According to the prevalence estimate in the present study, 4.53% of the general population of Hungary could be affected in these difficulties associated with high-level celebrity admiration.

This study has some limitations. Although the final prevalence rates were estimated on a weighted database, which is representative of the Hungarian adult population, due to the focus of the present study, data analysis concerning the methodological parts of scale development (i.e., factor structure, reliability, and validity testing) was conducted on an unweighted database (i.e., individuals who reported having a favorite celebrity and provided answers to at least 5 items of the CAS-7 items), which limits the generalizability of the findings to the whole population of Hungarian adults with a favorite celebrity. Moreover, future research is needed to confirm the psychometric appropriateness of the CAS-7 in other countries.

## Conclusions

In summary, the present study demonstrated the psychometric appropriateness of a short version of the widely used CAS, which allows for the identification and screening of individuals with high-level celebrity admiration who are also more likely to encounter mental health problems. The CAS-7 can possibly assist celebrity worship research and practice. Furthermore, this study is the first to provide prevalence estimates for high-level celebrity admiration (i.e., “celebrity worshipers”) using a general population sample. Findings indicated that only a small proportion of adults are affected, although the proportion of individuals with high-level celebrity admiration is higher among young adults.

## Supplementary material
