# Supplementary material for: Prevalence of celebrity worship: Development and application of the short version of the Celebrity Attitude Scale (CAS-7) on a large-scale representative sample
Source: J Behav Addict. 2024 Apr 18;13(2):463–72. doi: 10.1556/2006.2024.00019 (PMC11220816; doi:10.1556/2006.2024.00019)
Supplement: Supplementary file 1 [file jba-13-463-s001.docx]

**Zsila, Á. et al.: Prevalence of celebrity worship: Development and application of the short version of the Celebrity Attitude Scale (CAS-7) on a large-scale representative sample.** [**https://doi.org/10.1556/2006.2024.00019**](https://doi.org/10.1556/2006.2024.00019)

**Supplementary material**

**SM1**

*Detailed protocol for the selection for items of the final version of the CAS-7*

As the purpose of constructing a short version of the CAS (i.e., CAS-7) was to provide a brief and psychometrically sound assessment instrument that can measure healthy and problematic dimensions of celebrity admiration, a maximum of 2–3 items were considered per factor. Theoretical considerations were based on the key terms identified by McCutcheon et al. (2004) regarding the concept of each dimension. Specifically, items for the CAS-7 were selected to reflect the core components of each dimension with the greatest variability in content. Additionally, psychometric properties of the items were considered based on the study by Maltby et al. (2005) reproducing the original three-factor structure of the 23-item CAS. With regard to the ES factor, items containing “enjoyable”, “entertaining” (as a reference for “Entertainment”) and “group” (as a reference for “Social”) identified as key terms by McCutcheon et al. (2004) were considered, which also yielded high factor loadings (at or above 0.80; Maltby et al., 2005). Regarding the IP dimension, items comprising key terms indicating “Intense” feelings (“feel”) and “Personal” parasocial involvement (“special bond”) were considered, which could be more clearly differentiated from the BP dimension. “Obsessed” and “soul mate” were also identified as key terms by McCutcheon et al. (2004) constructing this dimension. However, items containing these terms had negative factor loadings on the BP dimension previously (Maltby et al., 2005), which is theoretically conflicting as the two problematic dimensions (i.e., IP and BP) are expected to have strong positive associations (Abraham et al., 2020; Wong et al., 2023). Finally, the key term “compelled” was considered for the BP dimension. The other item selected for this subscale had higher factor loading than the third item comprising this subscale in the study by Maltby et al. (2005), and also yielded a positive factor loading on the IP subscale.

**References**

Abraham, J., Ali, M. M., Andangsari, E. W., & Hartanti, L. E. P. (2020). Confirmatory factor analysis of celebrity worship, digital literacy, and nostalgia: Dataset of Indonesians. *Data in Brief*, *33*, 106417. <https://doi.org/10.1016/j.dib.2020.106417>

Maltby, J., Giles, D. C., Barber, L., & McCutcheon, L. E. (2005). Intense‐personal celebrity worship and body image: Evidence of a link among female adolescents. *British Journal of Health Psychology*, *10*(1), 17–32.

<https://doi.org/10.1348/135910704X15257>

McCutcheon, L. E., Maltby, J., Houran, J., & Ashe, D. D. (2004). *Celebrity worshippers: Inside the minds of stargazers*. Publish America.

Wong, M. M., McCutcheon, L. E., Rodefer, J. S., & Carter, K. (2023). Predicting the stalking of celebrities from measures of persistent pursuit and threat directed toward celebrities, sensation seeking and celebrity worship. *PLoS ONE*, *18*(3), e0281551. <https://doi.org/10.1371/journal.pone.0281551>

**Table S1**

*Fit indices for the Multiple Indicators Multiple Causes (MIMIC) models predicting celebrity admiration levels (Table 3)*

| **Model** | **df** | **χ^2^** | **CFI** | **TLI** | **RMSEA [ 90% CI]** | **SRMR** |
| --- | --- | --- | --- | --- | --- | --- |
| Model I | 28 | 67.225* | 0.983 | 0.974 | 0.043 [0.030–0.056] | 0.026 |
| Model II | 338 | 820.782* | 0.941 | 0.934 | 0.043 [0.039–0.047] | 0.045 |

^a^* *p* < 0.001

**Table S2**

*Fit indices for the latent profile analysis (LPA)*

| Classes | AIC | CAIC | BIC | SSABIC | Entropy | L-M-R test | *p* |
| --- | --- | --- | --- | --- | --- | --- | --- |
| 1 | 17921.42 | 17996.45 | 17986.45 | 17941.99 | – | – | – |
| 2 | 15973.37 | 16091.56 | 16075.56 | 16005.70 | 0.90 | 1927.78 | 0.000 |
| 3 | 15485.31 | 15646.66 | 15624.66 | 15529.40 | 0.83 | 494.75 | 0.007 |
| **4** | **15112.47** | **15316.98** | **15288.98** | **15168.31** | **0.86** | **381.66** | **0.000** |
| 5 | 14971.48 | 15219.15 | 15185.15 | 15039.08 | 0.87 | 215.11 | 0.119 |

^a^Classes = number of latent classes; AIC = Akaike information criterion; CAIC = bias-corrected Akaike information criterion; BIC = Bayesian information criterion; SSABIC = sample-size adjusted Bayesian information criterion; L–M–R test = Lo–Mendell–Rubin Adjusted Likelihood Ratio Test; *p* = *p*-value for the L–M–R Test.

^b^The final model is marked in bold.

**Figure S1**

*One-factor model of the CAS-7*

*
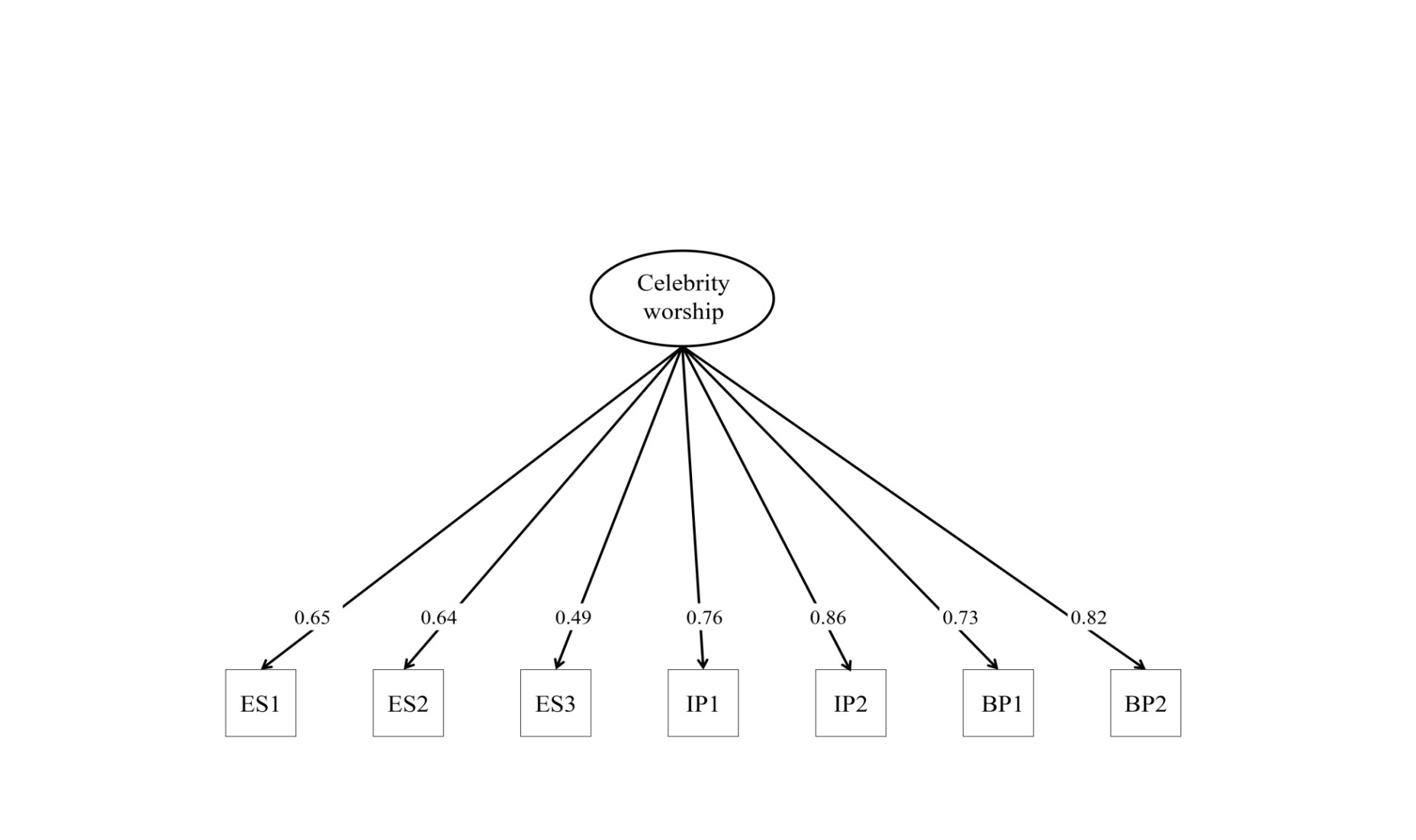
*

^a^The latent variable is represented in oval, while observed variables are represented in squares.

^b^ES = Entertainment–Social; IP = Intense–Personal, BP = Borderline–Pathological.

^c^Item numbers and their content are presented in Table 1.

^d^Standardized factor loadings are represented on the arrows (*p* < 0.001).

^e^Cronbach’s α = 0.88.

**Figure S2**

*Three-factor model of the CAS-7*

*
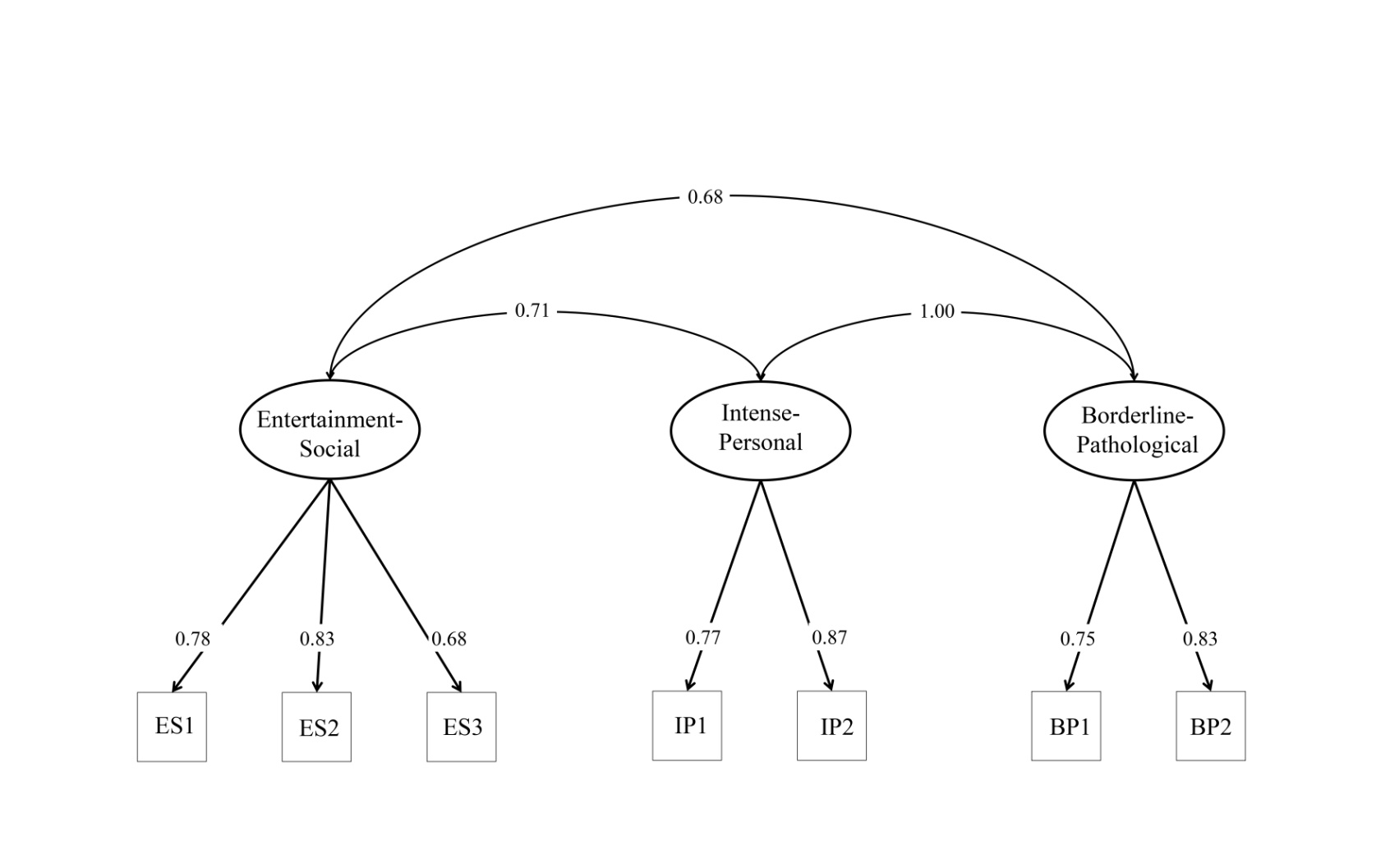
*

^a^Latent variables are represented in oval, and observed variables are represented in squares. ^b^ES = Entertainment–Social; IP = Intense–Personal, BP = Borderline–Pathological.

^c^Item numbers and their content are presented in Table 1.

^d^Standardized factor loadings are represented on single-headed arrows (*p* < 0.001). ^e^Correlation coefficients for the association between latent variables are presented on double-headed arrows (*p* < 0.001).

^f^Cronbach’s α = 0.81 for ES, α = 0.80 for IP, and α = 0.77 for BP.

**Table S3**

*Comparison of latent classes derived from the latent profile analysis based on mental health indicators*

|  | **Latent classes** | | | | **ANOVA** | |
| --- | --- | --- | --- | --- | --- | --- |
| **Variables**  **(*M, SD*)** | 1. | 2. | 3. | 4. | *F* | Cohen *d* |
| Depression, anxiety, and stress | 0.77 (1.27)a | 1.62 (1.70)b | 1.91 (1.89)b | 0.87 (1.07)a | 24.03* | 1–2: 0.57  1–3: 0.71  2–4: 0.53  3–4: 0.68 |
| Problematic Internet use | 11.78 (4.63)a | 14.73 (6.20)b | 16.95 (7.56)c | 11.60 (4.02)a | 30.03* | 1–2: 0.54  1–3: 0.82  2–3: 0.32  2–4: 0.60  3–4: 0.88 |

^a^* *p* < 0.001

^b^1 = low-level celebrity admiration class (*n* = 250; 32.51%); 2 = medium-level celebrity admiration class (*n* = 268; 34.85%); 3 = high-level celebrity admiration class (*n* = 119; 15.47%); 4 = high ES, low IPBP class (*n* = 132; 17.17%)
